# Self-initialized active contours for microscopic cell image segmentation

**DOI:** 10.1038/s41598-022-18708-5

**Published:** 2022-09-02

**Authors:** Asim Niaz, Ehtesham Iqbal, Farhan Akram, Jin Kim, Kwang Nam Choi

**Affiliations:** 1 Computer Science and Engineering Department, Chung-Ang University, Seoul, 06974 South Korea; 2 Department of Pathology and Clinical Bioinformatics, Erasmus Medical Center (EMC), 3015 Rotterdam, The Netherlands; 3 SecuLayer Inc., Seoul, 04781 South Korea

**Keywords:** Engineering, Mathematics and computing

## Abstract

Level set models are suitable for processing topological changes in different regions of images while performing segmentation. Active contour models require an empirical setting for initial parameters, which is tedious for the end-user. This study proposes an incremental level set model with the automatic initialization of contours based on local and global fitting energies that enable it to capture image regions containing intensity corruption or other light artifacts. The region-based area and the region-based length terms use signed pressure force (SPF) to strengthen the balloon force. SPF helps to achieve a smooth version of the gradient descent flow in terms of energy minimization. The proposed model is tested on multiple synthetic and real images. Our model has four advantages: first, there is no need for the end user to initialize the parameters; instead, the model is self-initialized. Second, it is more accurate than other methods. Third, it shows lower computational complexity. Fourth, it does not depend on the starting position of the contour. Finally, we evaluated the performance of our model on microscopic cell images (Coelho et al., in: 2009 IEEE international symposium on biomedical imaging: from nano to macro, IEEE, 2009) to confirm that its performance is superior to that of other state-of-the-art models.

## Introduction

Image segmentation has numerous applications because it is an essential building block for most image processing and computer vision tasks. Medical imaging, face recognition, pedestrian detection, etc., are some of these applications. Image segmentation helps divide an image into multiple non-overlapping regions to simplify them for further processing. The primary purpose of image segmentation is to simplify image representation in a meaningful way for image analysis. The quality of image segmentation has a significant impact on the reliability of the segmentation model. Artifacts such as intensity corruption in the images under observation greatly impact segmentation accuracy. Slight inaccuracies could propagate errors throughout the complete image processing chain. Hence, techniques that can manage these limitations are strongly desired.

Several methods are devised for the image segmentation, including region merging methods^1,2^, graph-based methods^3,4^, and the active contours methods (ACM)^5–14^. Kass et al. proposed ACM model, originally called snake model, based on energy minimization technique^5^. The principle of ACMs is to limit the evolving curve at the object boundaries by controlling inner and outer contour forces. Two drawbacks of original ACM are: (1) evolving contour finds it difficult to adapt to topological changes in image and (2) it is sensitive to initial condition. Level set-based active contour models need parameter initialization and the initial contour position (seeds), requiring technical skills from the end-user, making it an uphill task. Although these parameters values have a small effect on segmentation quality, expertise is still needed because it could lead to poor convergence. The seeds of initial contour should also be placed near the object of interest; otherwise, the accuracy and time cost could be compromised due to catching false contour lines.

**Figure 1:**
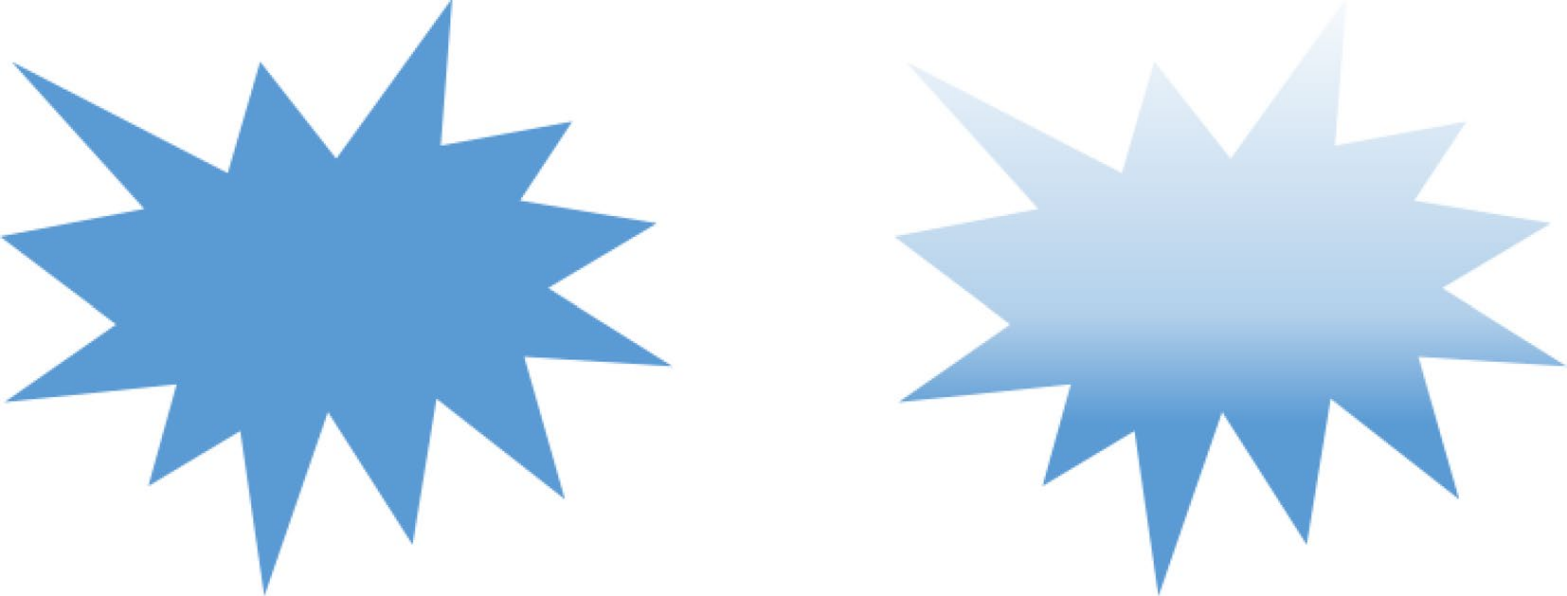
Example images: homogeneous image (left); inhomogeneous image (right).

ACMs are of two types:(1) global-region based and (2) local-region based. Both types have their pros and cons. For example, the local-region-based methods are good at segmenting local regions, and the global-region-based method can segment homogeneous areas efficiently. Mumford-Shah (M-S) model^6^ uses a set of contours C to partition different areas. However, it is challenging to minimize energy in the M-S model because the set C of low dimension is unknown. Chan and Vese proposed their region-based ACM^15^, assuming that the object of interest is of homogeneous intensities. Real images contain inhomogeneity due to bias conditions. Therefore, the C-V model worked well for the homogeneous images, but it is not suited for inhomogeneous image segmentation. To overcome this limitation, the Local Binary Fitted model was proposed based on the local intensity fitting term^7^. Zhang et al. proposed a local image fitting (LIF) model to segment local regions in an image^8^. C. Li proposed their energy function based on the local k-means clustering property and estimated bias field, responsible for the image inhomogeneity^9^. Figure 1 shows example images for homogeneous (free from intensity corruption) and inhomogeneous (with intensity corruption) types.

Wu et al. dealt with the inhomogeneity problem by introducing Retinex model^16^. Retinex incorporates a piece-wise variational level set with the bias field estimation and subsequent correction. Later, Wu et al. performed well on the homogenous and inhomogeneous images in their adaptive active contour model combined with the datafield^17^.

Region-based models are widely used for image segmentation due to their inherent noise filtering mechanism and ability to manage topological changes. However, their performance could be compromised if local fitting energy functionals are excluded. Image artifacts such as abrupt intensity variations within the same object of interest require the inclusion of a local fitting energy functional.

This study proposes an energy functional that comprises both the local and global fitting energy terms for inhomogeneous image segmentation. The proposed model includes a signed pressure force (spf) function in its area and length term to help it converge faster than other comparison methods. The inclusion of local and global fitting energies makes this method robust irrespective of image inhomogeneity. A hybrid energy functional is formulated and then penalized with SPF-based length and area terms. This work contributes to the level set-based ACM category of unsupervised segmentation.

This model is independent of the initial contour position. In addition, there is no need to initialize the parameters, unlike other models that require the end-users to initialize parameters. Inspired by the excluding seed method (ESM)^18^, the concept of walking particles is incorporated with the proposed method to assist with the self-initialization. The proposed model shows greater accuracy and is computationally less expensive than previous work.

The rest of this paper is organized as follows. “Section Proposed method” describes the level set formulation of the proposed model, and “Section Results” presents segmentation results, quantitative analysis, noise sensitivity analysis and computational cost evaluation followed by the ablation study where we presented two different *SPF* formulations. Subsequently, we have a discussion section and the final section concluding this manuscript.

## Proposed method

This section of the manuscript briefly explains the proposed model. Let us have an image *I*(*x*) in the *x*, *y* planes $R^2$. $\Omega$ represents the bounded open subset of the given domain, with domain boundary $\partial \Omega$. For initial contour position we use circular projection concept^18^ explained in the Discussion section of this manuscript. $C(s):[0,1]\rightarrow R^2$ is equivalent to the mathematical representation of a curve, dividing image *I*(*x*) into two distinctive regions, *inside*(*C*), and *outside*(*C*).

C-V^15^ bases their energy functional on the M-F^6^ model and proposes the following model:

1 $$\begin{aligned} E_{CV} (C,c_1,c_2)=\lambda _1\int _{\Omega }|I(x)-c_1|^2H_\varepsilon (\phi (x))dx +\lambda _2\int _{\Omega }|I(x)-c_2|^2(1-H)_\varepsilon (\phi (x)))dx +\mu \int _{\Omega }|\Delta H_\varepsilon (\phi (x))|^2dx+v\int _{\Omega }H_\varepsilon (\phi (x))dx \end{aligned}$$

where $\lambda _1$, $\lambda _2$, and *v* are the positive coefficients; $c_1$, and $c_2$ are are the average intensity means of the inner and outer regions of contour *C* in image *I*(*x*), mathematically given as

2 $$\begin{aligned} \begin{aligned} c_1=\dfrac{\int _{\Omega }I(x)H_\varepsilon \phi (x))dx}{\int _{\Omega }H_\varepsilon (\phi (x)))} \end{aligned} \end{aligned}$$

and

3 $$\begin{aligned} c_2=\dfrac{\int _{\Omega }I(x)(1-H_\varepsilon \phi (x)))dx}{\int _{\Omega }(1-H_\varepsilon (\phi (x)))} \end{aligned}$$

**Figure 2:**
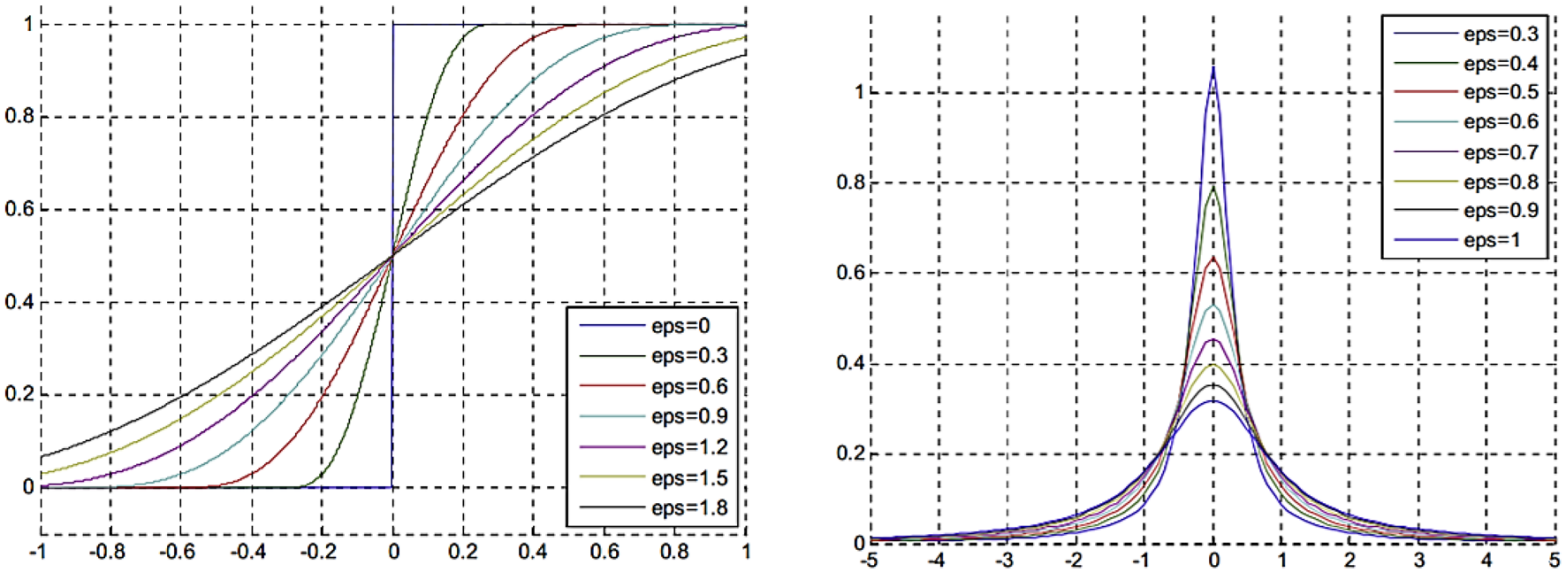
Effect of epsilon on (**a**) Heaviside function, and (**b**) Dirac delta function.

In these relations, $\varepsilon$ controls the smoothness of the $H(\phi )$, which is the smooth approximation of the Heaviside function. The effect of $\varepsilon$ on Heaviside function is illustrated in Fig. 2a.

4 $$\begin{aligned} H_\varepsilon (\phi (x))=\dfrac{1}{2}\left( 1+\dfrac{2}{\pi }arctan\left( \dfrac{\phi }{\varepsilon }\right) \right) \end{aligned}$$

The C-V^15^ model is a global region-based model that was originally designed based on the assumption that objects of interest have homogeneous intensities. The C-V^15^ model shows good performance while segmenting objects having homogeneous intensities. However, C-V^15^ fails to capture objects that have inhomogeneity due to corrupted intensity.

This work considers an image to be an approximation of the varying function of inhomogeneity. Variational level set with bias correction (VLSBC)^9^ suggested that an image under experiment contains some degree of intensity corruption. The image under experiment is equivalent to the bias field (*b*(*x*)), which is the region responsible for intensity corruption, true image (*J*(*x*)) that is free from noise and the additive noise (*n*(*x*)). The image under experiment can be described using the following mathematical expression:

5 $$\begin{aligned} I(x)= & {} b(x)J(x)+n(x),\quad x\in \Omega . \end{aligned}$$

6 $$\begin{aligned} J(x)= & {} \Sigma _{i=1}^{N}l_iM_i(\phi ) \end{aligned}$$

constitutes the constant approximation of an image that is free from inhomogeneity. K-means clustering, which is local clustering, is the minimization of

7 $$\begin{aligned} E\approx \int \left( \Sigma _{i=1}\int _{\Omega {i}}^{N}K_{\sigma } (x-y)|I(y)-b(x)c_i|^2dy\right) dx \end{aligned}$$

Using Heaviside function, (7) becomes

8 $$\begin{aligned} E=\int \left( \Sigma _{i=1}\int _{\Omega {i}}^{N}K_{\sigma } (x-y)|I(y)-b(x)m_i|^2M_i(\phi )dy\right) dx \end{aligned}$$

Here $N=2$, and $M_i$ accounts for the region member functions i.e $M_1=H(\phi )$, $M_2=1-H(\phi )$.

Taking the first derivative of (8), we obtain *b*(*x*), and $m_i$ as

9 $$\begin{aligned} b(x)=\Sigma _{i}^{N}\dfrac{{K_\sigma }*\left( I(x)m_iM_i(\phi )\right) }{{K_\sigma }*\left( c_i^2M_i(\phi )\right) } \end{aligned}$$

and

10 $$\begin{aligned} m_i=\int \dfrac{{K_\sigma }*\left( I(x)b(x)M_i(\phi )\right) }{{K_\sigma }*\left( {b(x)}^2M_i(\phi )\right) } \end{aligned}$$,

respectively.

VLSBC^9^ is robust to initialization and guarantees the smoothness of the bias field over the data term.

The proposed model, inspired by^19,20^ segments inhomogeneous images by combining local and global fitting energies as

11 $$\begin{aligned} E_{proposed}=E_{LGFE}(\phi )+\mu L_{spf}(\phi )+vA_{spf}(\phi ) \end{aligned}$$

where

12 $$\begin{aligned} L_{spf}(\phi )=\int _{\Omega }spf(I)\delta _\varepsilon (\phi )|\Delta \phi |dx \end{aligned}$$,

and

13 $$\begin{aligned} A_{spf}(\phi )=\int _{\Omega }spf(I)H_\varepsilon (\phi )dx \end{aligned}$$

are the region-based length and region-based area terms, respectively. Even though the terms $L_{spf}(\phi )$ and $A_{spf}(\phi )$ are inspired from Akram et al.^19^, they are different in a way that we have not appended membership function in the SPF functions of each of these terms in contrast to the Akram et al.^19^. There is no need to append the membership function within the SPF of these region-based terms in the proposed model (this can be verified in the ablation study section) because it is already added with (9) and (10). $E_{LGFE}$ is the local global fitting energy model, defined as

14 $$\begin{aligned} E_{LGFE}(\phi )=\int {\left( (I(x)-I_{bLFI})(I(x)-I_{GFI})\right) }dx \end{aligned}$$

where $I_{bLFI}$ and $I_{GFI}$ are the local image fitted and global image fitted models, respectively, and are defined as follows:

15 $$\begin{aligned} I_{bLFI}= & {} b(x)(m_1M_1+m_2M_2) \end{aligned}$$

16 $$\begin{aligned} I_{GFI}= & {} c_1M_1+c_2M_2 \end{aligned}$$

In (12) and (13), *spf* is a signed pressure force function, defined as

17 $$\begin{aligned} spf(I)=\dfrac{I(x)-I_{GFI}(x)}{max(|I(x)-I_{GFI}(x)|)} \end{aligned}$$

By calculus of variation^21^, (11) minimizes to

18 $$\begin{aligned} \dfrac{\partial \phi }{\partial {t}}= \delta _\varepsilon (\phi )(I(x)-I_{bLFI}(x))(c_1-c_2) +\delta _\varepsilon (\phi )(I(x)-I_{GFI}(x))(m_1-m_2) +\left( \mu div\left( spf(I)\dfrac{\Delta (\phi )}{|\Delta (\phi )|}\right) \right) \delta _\varepsilon (\phi )-vspf(I)\delta _\varepsilon (\phi ) \end{aligned}$$

where $\delta _\varepsilon (\phi )$ is Dirac delta function, defined as

19 $$\begin{aligned} \delta _\varepsilon (\phi )=\dfrac{\varepsilon }{\pi (\phi ^2 + \varepsilon ^2)} \end{aligned}$$

The effect of $\varepsilon$ on the Dirac delta function is illustrated by Fig. 2b. $\varepsilon$ is a constant that controls the width of the Dirac delta function. $c_i$ and $m_i$ are the global and local intensity means defined by (2), (3), and (10), respectively. The global intensity means are computed under the assumption that the image contains homogeneous regions. Therefore, the inclusion of the bias field *b*(*x*) is included in the local intensity means to ensure efficient contour evolution over inhomogeneous images as well.

The mathematical value of *spf*(*I*) is $[-1,1]$ inside and outside the contour. *spf*(*I*) modulates the pressure force sign within the region of interest (ROI), such that it attracts the contour if outside, and expands the contour if inside the ROI. It helps to obtain a smooth version of the gradient descent flow.

**Figure 3:**
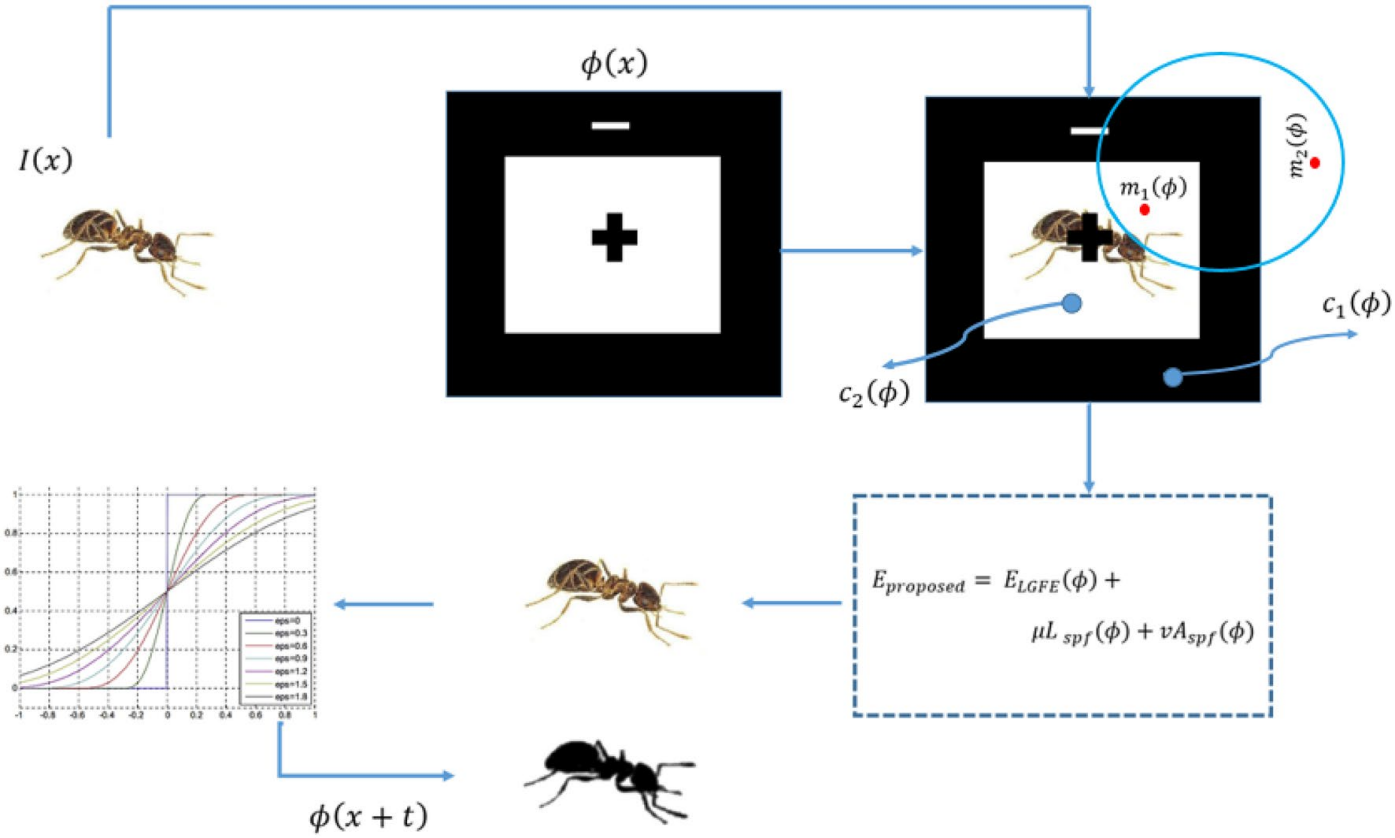
Graphical representation of the proposed algorithm.


A graphical presentation of the proposed model is presented in Fig. 3. The proposed model can automatically estimate the default parametric values depending on the characteristics of the object of interest. Furthermore, if the images of a new patient are fed into the model, this will automatically adjust the default values of the initial parameters, thereby eliminating the inconvenience of different results. The initial level set function of the proposed model is defined as

20 $$\begin{aligned} \phi _{x,t=0}={\left\{ \begin{array}{ll} -p, &{} x\in \Omega _0-\partial \Omega _0\\ 0, &{}x \in \partial \Omega _0\\ p, &{} x\in \Omega -\partial \Omega _0 \end{array}\right. } \end{aligned}$$

where *p* is a positive constant, i.e., $p>0$, $\Omega$ represents the image domain; $\Omega _0$ represents the initial contour inner region; $\partial \Omega _0$ is the initial contour. Later stages of the proposed algorithm are listed below.

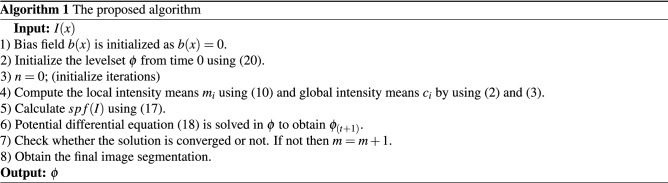


## Results

In this section, the proposed model is compared with other level set models on synthetic and real images. All experiments were conducted using MATLAB 2018 on a PC with Windows 10 and an Intel $\circledR$ Core$^\mathrm{TM}$ i7, 3.60 GHZ and 8192 MB RAM.

**Figure 4:**
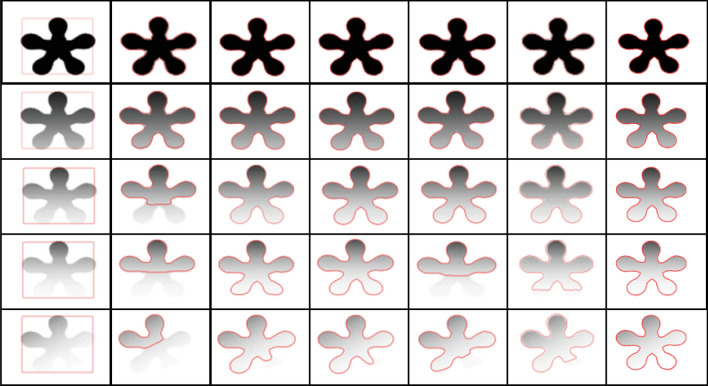
Comparison of proposed model with other models on the same image with five different levels of inhomogeneity: (col 1) input image with initial contour; (col 2) C-V^15^; (col 3) LBF^7^; (col 4) LIF^8^; (col 5) VLSBC^9^; (col 6) Zhang et al.^10^; (col 7) proposed model.

Figure 4 presents the experimental results on a synthetic image with five different levels of inhomogeneity in increasing order from top row to the bottom. The first column shows input images, followed by a comparison of different models with the proposed model. For the first two levels, all the methods captured the region of interest to the full extent. However, the global region-based active contour models struggle to evolve as the inhomogeneity level increases correctly. On the other hand, the local-region-based models performed well as compared to the global-region-based methods. The proposed model, containing local and global region-based terms appended with the bias field and region-membership functions, captures ROI irrespective of the image inhomogeneity levels.

**Figure 5:**
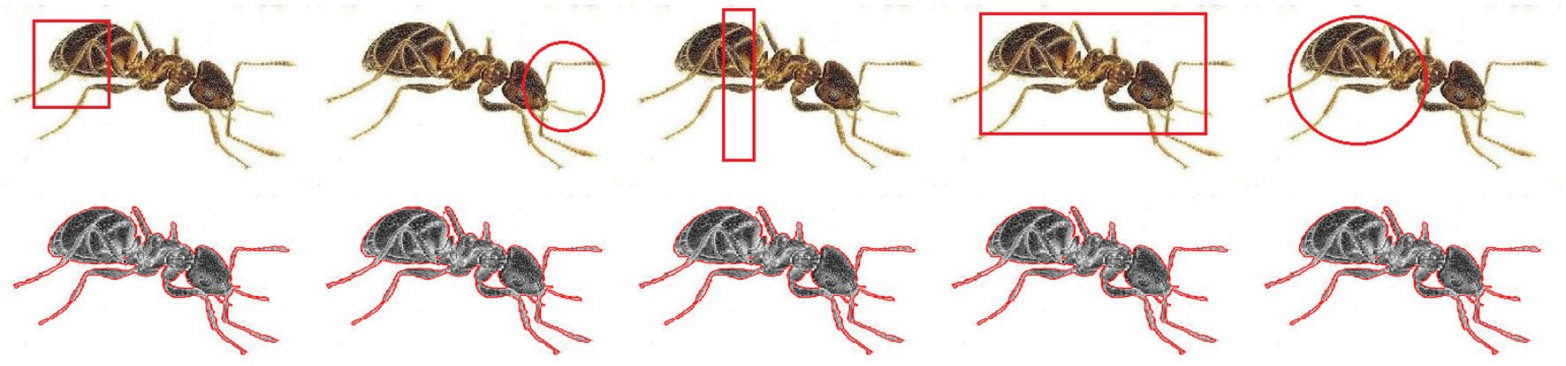
Segmentation results with contours of different shapes and sizes at different locations on the input image. First row: input image with different contours; second row: associated results.

Figure 5 showcases the segmentation results confirming the independence of our model of the initial contour position. For this purpose, we used contours of different shapes and sizes over the same image and observed the corresponding effect. It is evident that the position of the initial contour has no or negligible effect on segmentation.

The performance of different active contour methods is compared to the proposed approach on multiple synthetic and real images.

### Synthetics examples

**Figure 6:**
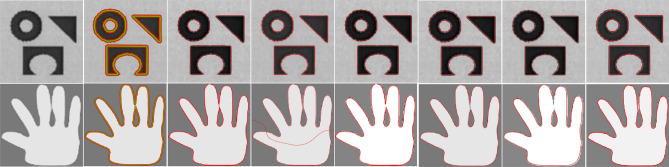
Results of the proposed model in comparison with those of other models on synthetic images: (col 1) input image; (col 2) C-V^15^; (col 3) LBF^7^; (col 4) LIF^8^; (col 5) VLSBC^9^; (col 6) Zhang et al.^10^; (col 7) FRAGL ; (col 8) proposed model.

Figure 6 represents a comparison of different ACMs over synthetic example images. The original images are shown in the first column, followed by the results produced by C-V^15^, LBF^7^, LIF^8^, VLSBC^9^, Zhang et al.^10^, and FRAGL^11^, respectively. The top row of Fig. 6 exhibits almost similar segmentation accuracy for all the comparison methods. The second row of Fig. 6 is a computer-generated image of fingers. Although C-V^15^ produced smooth contours around the boundaries of the fingers, it did not fully distinguish the middle and the ring fingers. This limitation is there because the C-V^15^ only considers the global statistics and not the local statistical information during contour evolution. Utilizing LIF energy, the LIF^8^ differentiated the middle and ring finger boundaries, but false contours compromised the segmentation accuracy of this model. LBF^7^ and VLSBC^9^ showed similar results, whereas the Zhang et al.^10^ model gave comparatively less segmentation accuracy than all the other comparison methods.

Table 1 presents the statistical data for CPU time (s) and iteration counts measured for the mentioned methods for the individual images in The computational cost of the proposed model is significantly less than the previous methods.

**Table 1:** CPU time (s) and number of iterations consumed for segmentation results of Fig. 6.

Row No.	Methods													
	C-V^15^		LBF^7^		LIF^8^		VLSBC^9^		Zhang et al.^10^		FRAGL^11^		Proposed	
	CPU time (s)	Iterations	CPU time (s)	Iterations	CPU time (s)	Iterations	CPU time (s)	Iterations	CPU time (s)	Iterations	CPU time (s)	Iterations	CPU time (s)	Iterations
1	1.98	100	7.44	500	0.91	80	0.88	7	3.46	50	1.08	10	1.09	2
2	5.02	500	7.68	500	1.93	500	3.41	36	4.75	50	1.79	50	1.47	5

### Real examples

**Figure 7:**
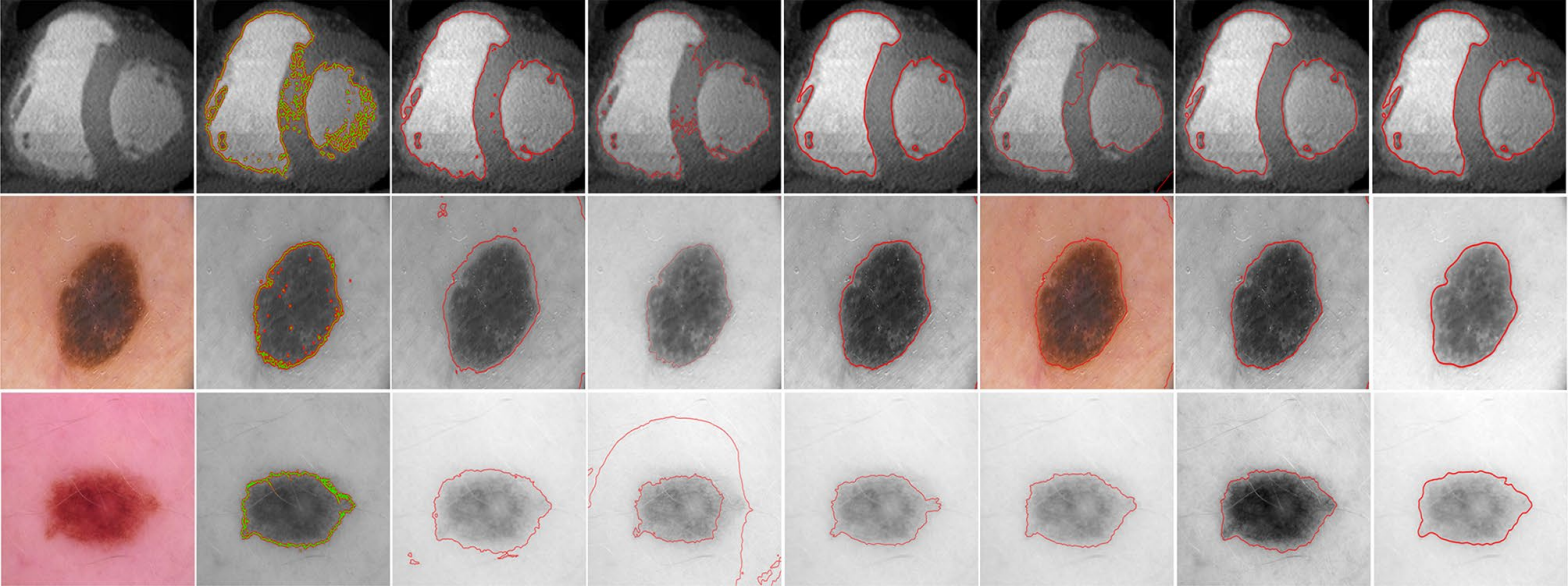
Results of proposed model in comparison with those of other models on real medical images: (col 1) input image; (col 2) C-V^15^; (col 3) LBF^7^; (col 4) LIF^8^; (col 5) VLSBC^9^; (col 6) Zhang et al.^10^; (col 7) FRAGL^11^ ; (col 8) proposed model.

Figure 7 is a comparison of multiple methods on real medical images. The three rows of this figure have a medical image, a dermoscopic image from the PH2 database^12^, and a skin lesion image from the Skin-Cancer-MNIST-HAM10000 database^13^, respectively.

The segmentation results produced by the FRAGL^11^ method and the proposed method are almost the same. The C-V^15^ model failed to manage the inhomogeneity in images and produced noisy segmentation. The LBF^7^ and LIF^8^ models captured the ROI, but the appearance of false contours compromised the segmentation accuracy.

Table 2 presents the statistical data for CPU time (s) and iteration counts measured for the mentioned methods for individual images in Fig. 7. The CPU time complexity of our model is significantly less compared to the previous models.

**Table 2:** CPU time (s) and number of iterations consumed for segmentation results of Fig. 7.

Row No.	Methods													
	C-V^15^		LBF^7^		LIF^8^		VLSBC^9^		Zhang et al.^10^		FRAGL^11^		Proposed	
	CPU time (s)	Iterations	CPU time (s)	Iterations	CPU time (s)	Iterations	CPU time (s)	Iterations	CPU time (s)	Iterations	CPU time (s)	Iterations	CPU time (s)	Iterations
1	3.44	100	5.71	30	4.80	500	2.81	50	1.43	50	0.99	10	0.91	2
2	4.01	80	5.79	35	5.06	500	3.31	50	1.69	60	1.11	12	1.01	3
3	2.88	50	4.37	30	6.08	400	3.98	60	2.05	50	2.09	15	1.07	3

### Quantitative analysis

This section presents the results for the proposed model in comparison with the LIF^8^, VLSBC^9^, Zhang et al.^10^, and FRAGL^11^ methods against ground-truths. To validate the superiority of our model, we measured the segmentation accuracy metric for the microscopic cell image dataset^22^ as:

21 $$\begin{aligned} Accuracy=\dfrac{TP+TN}{TP+FP+TN+FN}, \end{aligned}$$

where *TP*, *TN*, *FP*, and *FN* account for *truepositive*, *truenegative*, *falsepositive*, and *falsenegative*, respectively. The segmentation accuracy of the proposed method is the highest, making it the most efficient of all the comparison methods.

To further add transparency to the experiments, we extended our evaluation to measure the Die Index, Jaccard similarity index (Jaccard index), and contour matching score (bfscore). All of these metrics are calculated for the proposed method and the comparison methods. The range of these metrics lies within [0, 1], where a value closer to 1 indicates a more precise segmentation.

**Figure 8:**
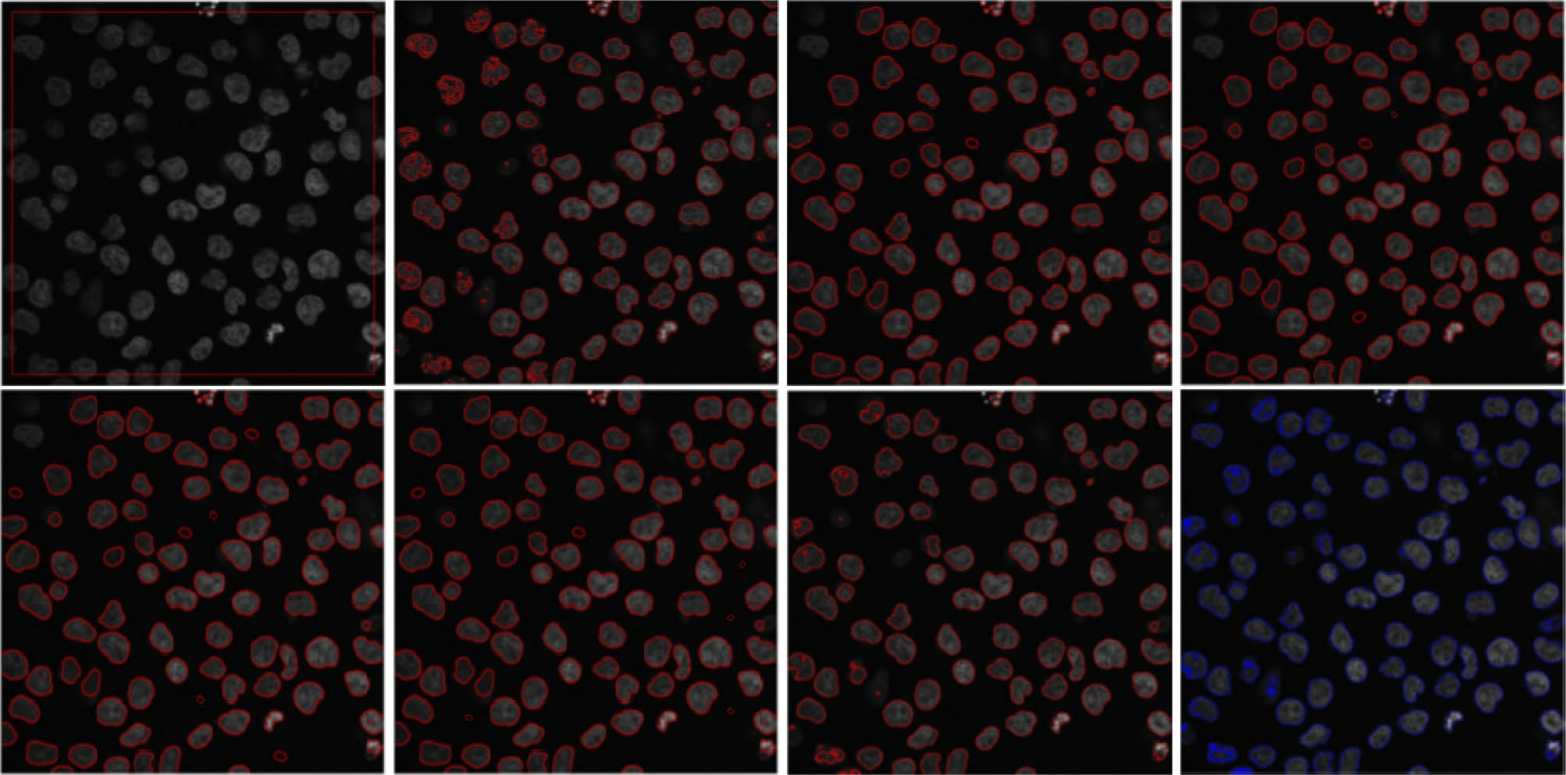
Top row: input image with initial contour, segmentation results for LIF^8^, VLSBC^9^ and segmentation results for Zhang et al.^10^, respectively. Second row: segmentation results for Akram et al.^19^, Akram et al.^20^, FRAGL^11^, and the proposed method, respectively.

**Figure 9:**
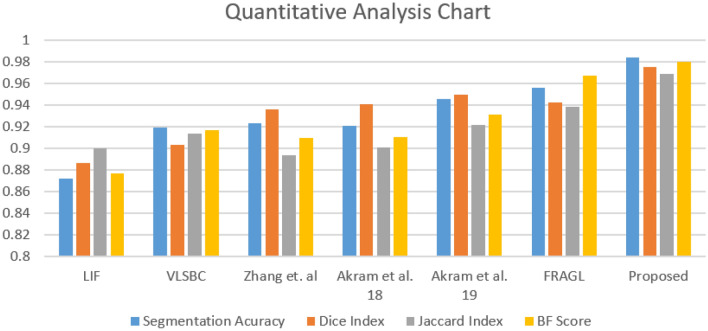
Quantitative analysis chart showing a graphical illustration of segmentation accuracy, Dice index, Jaccard index and BF score matrices.

The Dice index is usually called an overlap index, and it is measured by overlapping the segmentation and ground truth masks by placing one on top of the other. The value of the Dice index shows to what degree the segmentation result is comparable to the actual result. The Dice index is measured using the segmentation result (SR) and ground truth (GT). The formula used to calculate the Dice Index is as follows:

22 $$\begin{aligned} Dice (SR, GT) \dfrac{2|S_r \cap S_g| }{|S_r| + |S_g|} \end{aligned}$$

where $S_r$ and $S_g$ define the segmented results and the actual ground truths, respectively. We then calculate the Jaccard index as:

23 $$\begin{aligned} Jaccard Index (SR, GT) = \dfrac{|S_r \cap S_g|}{|S_r + S_g|} \end{aligned}$$

Likewise, the contour matching score (BF score) helps estimate how close the boundary of the segmented region is to the ground truth boundary. The BF score is measured as:

24 $$\begin{aligned} BF(SR, GT) = \dfrac{\alpha _1 \alpha _2}{\alpha _1 + \alpha _2} \end{aligned}$$

$\alpha _1$ is the proportion of the number of points on the boundary of the SRs that are sufficiently close to the boundary of the GT to the length of the boundary of SR.

$\alpha _2$ is the proportion of the number of points on the boundary of the GT that are sufficiently close to the boundary of the SRs to the length of the GT boundary. The range of the BF score is [0,1]. The higher the BF score, the better the segmentation quality.

These quantitative comparisons were conducted for all models to measure the segmentation accuracy, Dice index, Jaccard index, and BF score over the microscopic images^22^ dataset of dermoscopic images. Figure 8 is the visual presentation of segmentation results for LIF^8^, VLSBC^9^, Zhang et al.^10^, Akram et al.^19^, Akram et al.^20^, FRAGL^11^, and the proposed model, and Fig. 9 presents the quantitative illustration of various performance metrics for all the comparison methods.

All the image segmentation evaluation techniques prove that the proposed active contour model achieves the highest Dice index, Jaccard index, and contour matching score (BFscore) compared with the previous related models.

### Noise sensitivity evaluation

This section presents a noise sensitivity analysis of the proposed model using the Jaccard Similarity Index (JSI). JSI helps compare segmentation accuracy when the input images are corrupted with noise. We added different levels of artificial salt & pepper and Gaussian noise to the input images to measure the robustness of various methods, including the proposed model. The mathematical relation of the JSI is:

25 $$\begin{aligned} JSI (X, Y )= \dfrac{|X \cap Y|}{|X \cup Y|}, \end{aligned}$$

where *X* and *Y* represent the segmentation result and the ground truth, respectively.

**Figure 10:**
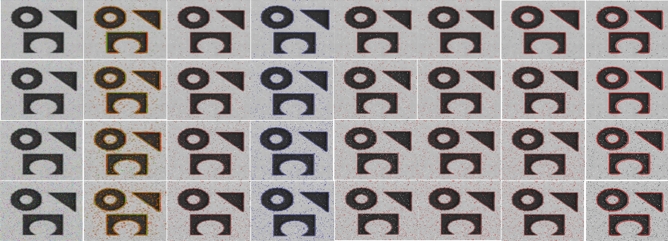
Col (1): Input image corrupted with Salt & Pepper noise levels (0.01, 0.02, 0.03, 0.04, 0.05), Col(2): Segmentation results of (col 2) C-V^15^; (col 3) LBF^7^; (col 4) LIF^8^; (col 5) Adaptive^17^; (col 6) Retinex^16^; (col 7) FRAGL ; (col 8) proposed model.

**Figure 11:**
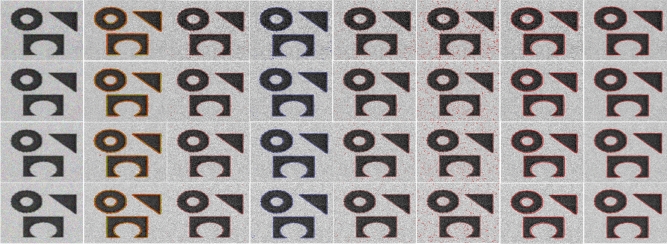
Col (1): Input image corrupted with Guassian noise levels (0.01, 0.02, 0.03, 0.04, 0.05), Col(2): Segmentation results of (col 2) C-V^15^; (col 3) LBF^7^; (col 4) LIF^8^; (col 5) Adaptive^17^; (col 6) Retinex^16^; (col 7) FRAGL ; (col 8) proposed model.

Figures 10 and 11 show segmentation results of different ACMs and the proposed method when the image is corrupted with salt & pepper and Gaussian noises, respectively. The input image we considered representing noise sensitivity evaluation in the manuscript is of average complexity. The reason is that most models, irrespective of their genres, perform better on such images.

**Figure 12:**
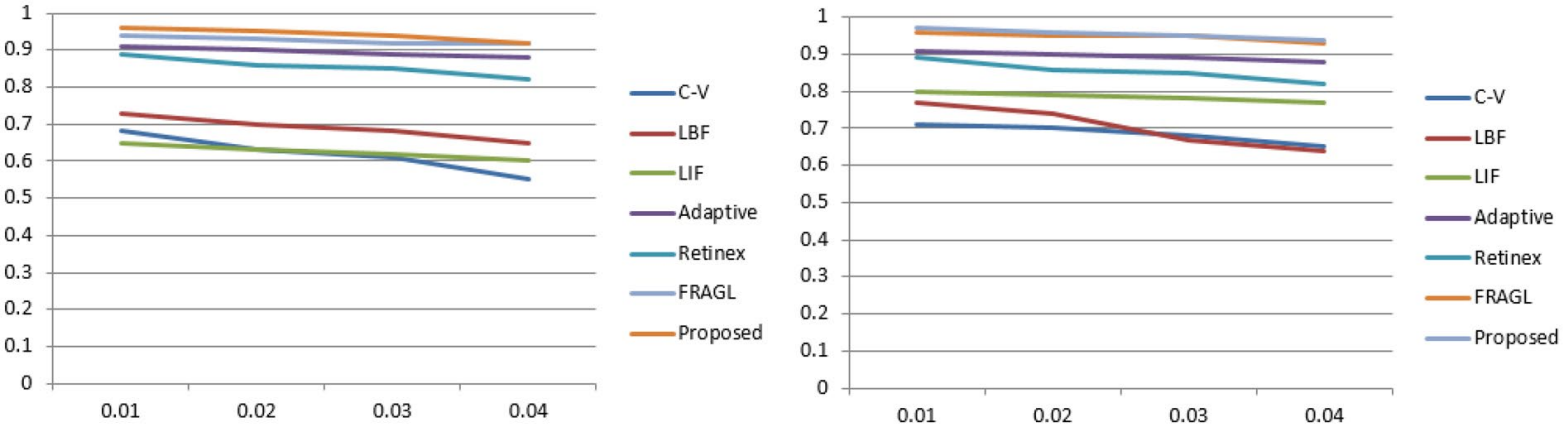
JS values for Figs. 10 and 11 are represented by (left) and (right), respectively.

Both Figs. 10 and 11 show that all the ACM methods in the experiment have captured the ROI well; however previous methods took longer than the proposed model to converge. JS values of all the in-comparison methods are calculated by comparing segmentation results with ground-truth using (25). The respective accuracy chart for both the figures is shown in Fig. 12. It is clearly evident that the proposed model has higher JS values nearly equal to 1, confirming its robustness to different noise levels.

### Computational cost evaluation

**Figure 13:**
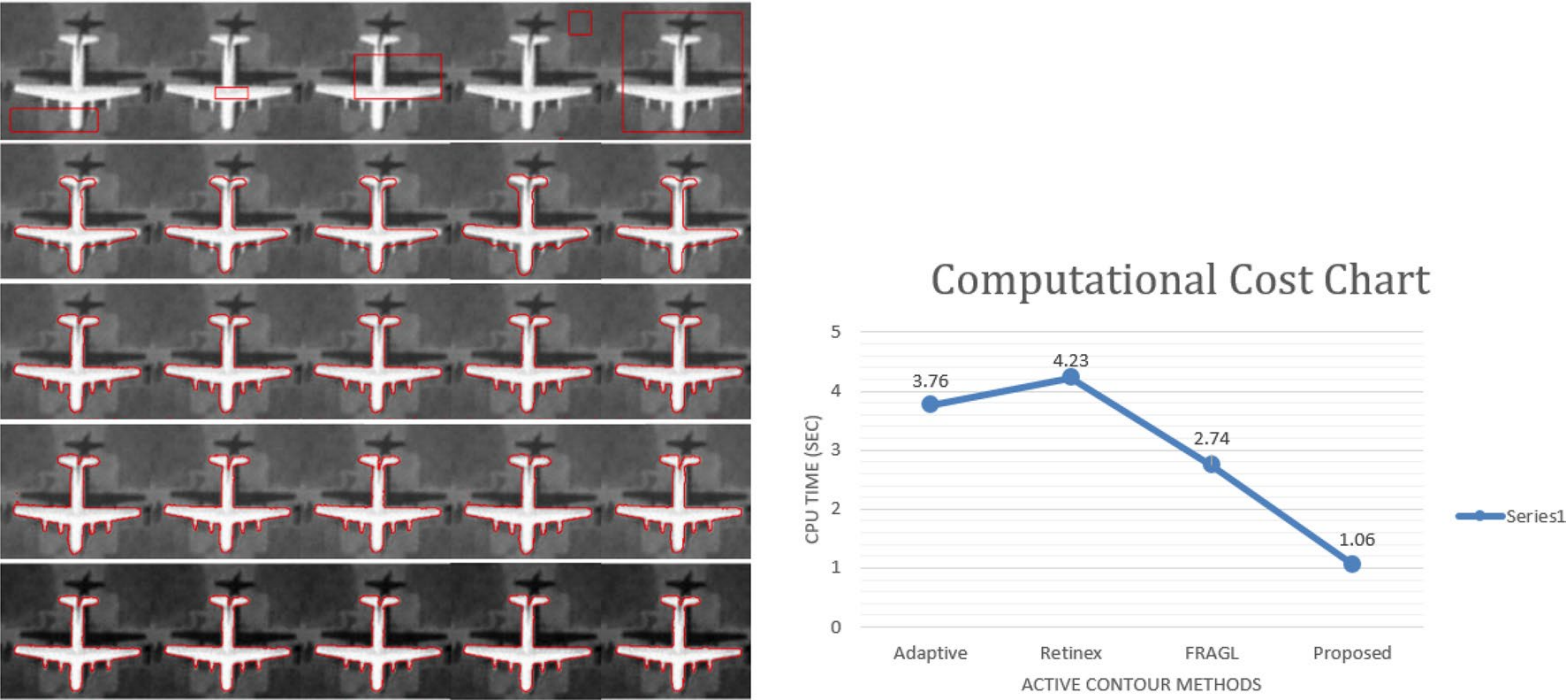
Right Image: Row (1): Input image with initial contours pf different shapes at different positions; Row (2) Adaptive^17^; Row (3) Retinex^16^; Row (4) FRAGL^11^; Row (5) proposed model. Left Image: Computational Cost Chart.

This section presents one of the several images we tested various methods on to evaluate computational cost or the CPU time. The presentation image is of an airplane along with its shadow. We compared the performance of Retinex^16^, Adaptive^17^, FRAGL^11^, and the proposed model in terms of CPU time. All in-comparison methods captured the object of interest, the airplane, disregarding its shadow. We noted the computational time for each method to evolve over the object of interest until the contour fully fits it. The computational cost chart is presented in Fig. 13, along with the airplane image segmentation results. Adaptive^17^ model took 3.76 s to converge to the final segmentation, which is the second-best amongst competitors, while the Retinex^16^ model took 4.23 s to segment the object of interest. FRAGL^11^ model shows the performance of 3.74 s, slightly slower than the proposed model, which is just 1.06 s.

### Ablation study

**Figure 14:**
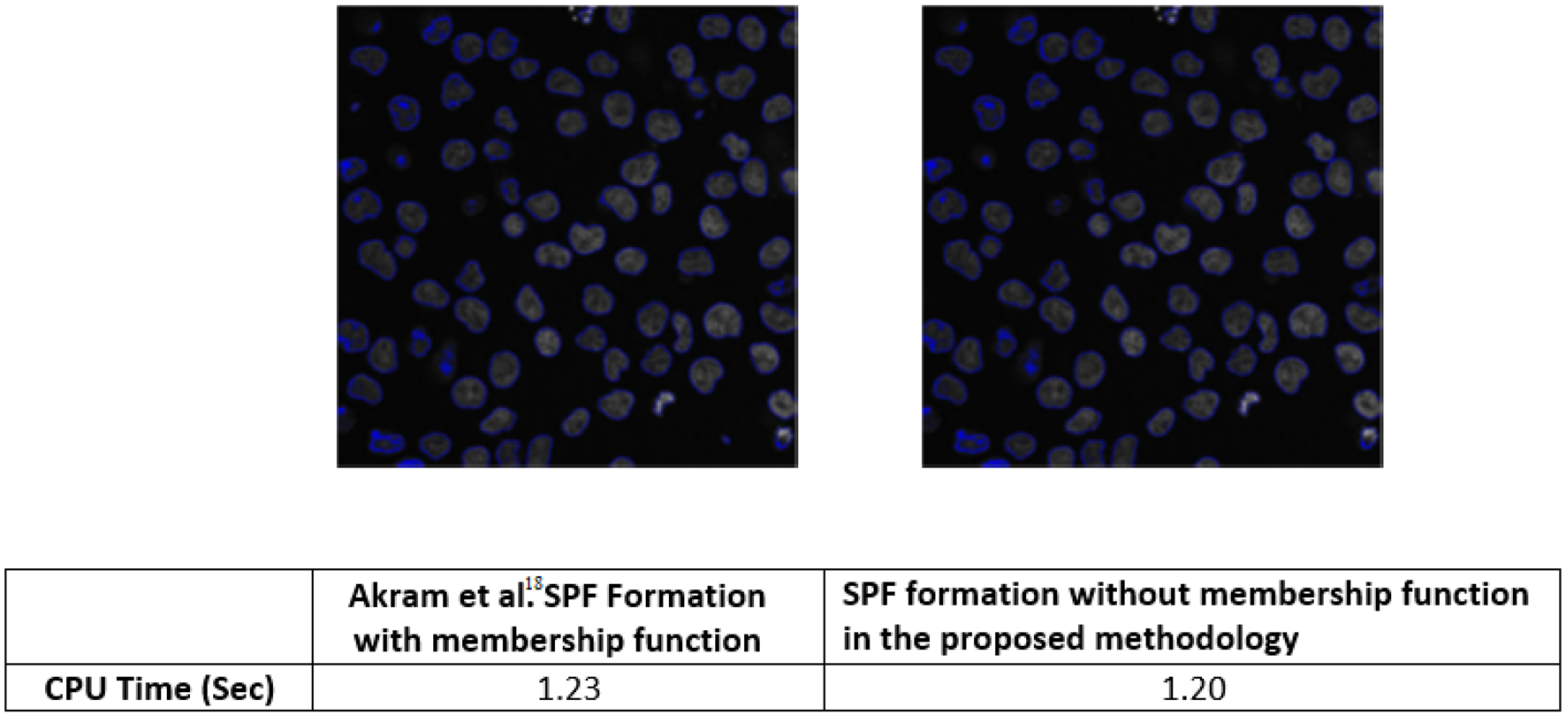
Segmentation results against time complexity (**a**) SPF with the Akram et al.^19^ formation (**b**) SPF formation with out membership function in the proposed methodology.

This section presents the ablation study where we studied the impact of different terms of the proposed model on its overall performance for the microscopic image^22^. Figure 14 presents the line graph for segmentation accuracy concerning the full energy functional of the proposed model, removing both of the region-based terms, removing length term and removing area term, respectively.

**Figure 15:**
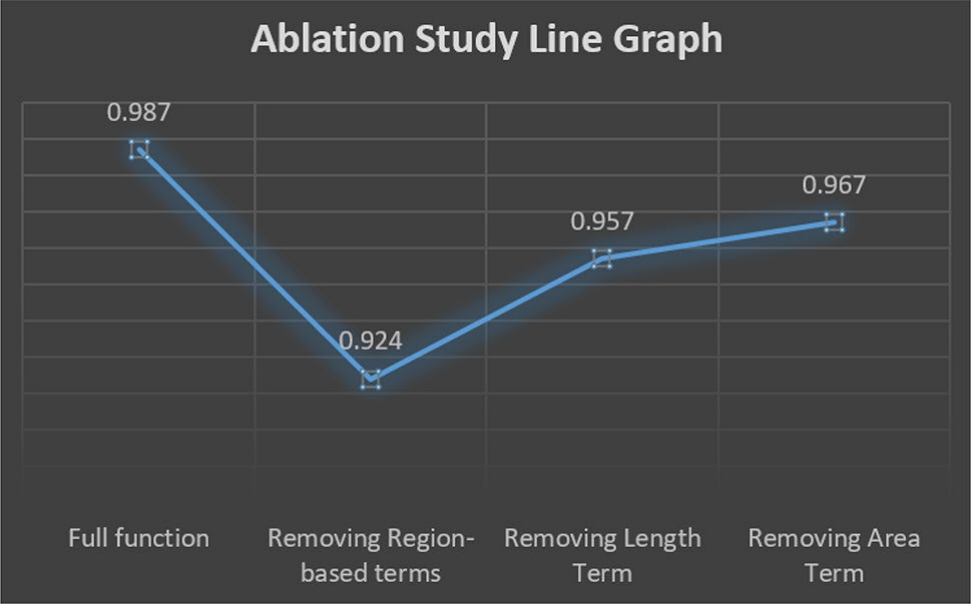
Ablation study over microscopic images^22^ database by removing different terms from the proposed function.

Furthermore, we also observed the impact of including membership function with the SPF function of both the region-based terms as in the Akram^19^ model. The results show that we achieve a similar type of results with the only difference that it adds to the time complexity by $3\%$. This difference does not look great if we take the computations for one image; however, at a large scale, this difference is worth expensive. Therefore, we opt not to append membership function with the SPF of both the region-based terms. Figure 15 presents results from both the formations against time complexity of each.

## Discussion

Level set-based image segmentation models require parameter initialization that is randomly selected from the literature. This requirement compromises such models’ performance if the end-user does not select suitable parameters. Circular Projection (CP) assists with estimating the converging or diverging configuration, which helps select seeds inside the object of interest. Inspired by ESM^18^, the CP is defined as:

26 $$\begin{aligned} P^+_{x, y}(\theta )=\Sigma _{x, y}{\left\{ \begin{array}{ll} (proj_{v_{R}(\theta )} V{x, y})^2 &\quad {if} proj_{v_{R}(\theta )} V{x, y} > 0,\\ v_{x, y} {\in W^z_{x, y}} &\quad v_{x, y} {\in W^z_{x, y}}\\ 0, &\quad otherwise \end{array}\right. } \end{aligned}$$

and

27 $$\begin{aligned} P^-_{x, y}(\theta )=\Sigma _{x, y}{\left\{ \begin{array}{ll} (proj_{v_{R}(\theta )} V{x, y})^2 &\quad {if} proj_{v_{R}(\theta )} V{x, y} < 0,\\ v_{x, y} {\in W^z_{x, y}}&\quad v_{x, y} {\in W^z_{x, y}}\\ 0, &\quad otherwise \end{array}\right. } \end{aligned}$$

where $V_{x, y}$ is a discrete vector filed $W^z_{x, y}$ having a window of size *z* with its center at point *x*, *y* of the discrete vector field. $v_R \theta =({}^{cos\theta } {}_{sin\theta }), 0\le \theta \le \pi$.

Most image segmentation models belong to the supervised category requiring large and densely annotated datasets. However, there is a scarcity of medical datasets as researchers have access to limited samples. And the performance of such supervised models is compromised if they are trained on fewer images which could be dangerous in medical applications. The proposed model does not belong to the unsupervised category of the image segmentation literature that does not require large datasets to produce superior segmentation results.

## Conclusion

This paper presents a novel method that is based on both the local and global fitting models and is smoothed by Gaussian filtering. This method segments homogeneous as well as inhomogeneous images. The bias field is incorporated with the local fitting model to ensure contour evolution over inhomogeneous regions. The Gaussian kernel provides contour smoothness over object boundaries after each iteration. The proposed model successfully and efficiently dealt with bias conditions and outclassed the other segmentation methods, as confirmed by the results and quantitative analysis sections. In the future, we will evaluate the proposed model on additional types of images.

## Data Availability

The microscopic cell image dataset^22^ analysed for the quantitative study is available at Murphy-Lab. Rest of the images analysed/generated in this manuscript are available from the corresponding author on reasonable request.
